# Marker Development for Differentiation of *Fusarium oxysporum* f. sp. *Niveum* Race 3 from Races 1 and 2

**DOI:** 10.3390/ijms22020822

**Published:** 2021-01-15

**Authors:** Owen Hudson, Sumyya Waliullah, James C. Fulton, Pingsheng Ji, Nicholas S. Dufault, Anthony Keinath, Md Emran Ali

**Affiliations:** 1 Department of Plant Pathology, University of Georgia, Tifton, GA 31793, USA; Owen.hudson@uga.edu (O.H.); Sumyya.Waliullah@uga.edu (S.W.); pji@uga.edu (P.J.); 2 Department of Plant Pathology, University of Florida, Gainesville, FL 32611, USA; pcvgt@ufl.edu (J.C.F.); nsdufault@ufl.edu (N.S.D.); 3 Department of Plant and Environmental Sciences, Clemson University, Charleston, SC 29414, USA; tknth@clemson.edu

**Keywords:** *Fusarium oxysporum* f. sp. *niveum*, comparative genomics, race differentiation, polymerase chain reaction, watermelon

## Abstract

Fusarium wilt of watermelon, caused by *Fusarium oxysporum* f. sp. *niveum* (FON), is pathogenic only to watermelon and has become one of the main limiting factors in watermelon production internationally. Detection methods for this pathogen are limited, with few published molecular assays available to differentiate FON from other formae speciales of *F. oxysporum*. FON has four known races that vary in virulence but are difficult and costly to differentiate using traditional inoculation methods and only race 2 can be differentiated molecularly. In this study, genomic and chromosomal comparisons facilitated the development of a conventional polymerase chain reaction (PCR) assay that could differentiate race 3 from races 1 and 2, and by using two other published PCR markers in unison with the new marker, the three races could be differentiated. The new PCR marker, FNR3-F/FNR3-R, amplified a 511 bp region on the “pathogenicity chromosome” of the FON genome that is absent in race 3. FNR3-F/FNR3-R detected genomic DNA down to 2.0 pg/µL. This marker, along with two previously published FON markers, was successfully applied to test over 160 pathogenic FON isolates from Florida, Georgia, and South Carolina. Together, these three FON primer sets worked well for differentiating races 1, 2, and 3 of FON. For each marker, a greater proportion (60 to 90%) of molecular results agreed with the traditional bioassay method of race differentiation compared to those that did not. The new PCR marker should be useful to differentiate FON races and improve Fusarium wilt research.

## 1. Introduction

*Fusarium oxysporum* f. sp. *niveum* (FON) is the causal agent of Fusarium wilt of watermelon and a common limiting factor for watermelon production worldwide [1,2,3,4,5]. FON is a soilborne ascomycete fungus and one of at least 106 formae speciales within the *Fusarium oxysporum* species complex [6]. As with all formae speciales of *F. oxysporum*, FON has three types of spores: single-celled microconidia, multi-celled macroconidia, and over-wintering chlamydospores. The long-lasting survivability of chlamydospores demonstrates the challenge of combating this pathogen using crop rotation and chemical fumigants [5,7,8,9]. In addition, diagnosis based on spore morphology cannot be done to the forma specialis level due to the likeness of spores between formae speciales and other *Fusarium* species [10,11,12].

Fusarium wilt can be identified in the field by observing a single side or vine of the watermelon plant wilting with the rest of the plant being unaffected [13]. Wilting is due to the accumulation of microconidia in the plant’s xylem tissue and the defense response of the plant to form tyloses, a mechanism by which the plant suppresses fungal growth [5,14,15]. The vasculature of the watermelon plant turns red/brown before the infection spreads to the rest of the plant which is a key diagnostic feature in the field [16,17,18,19]. Often, FON infections occur early in the growth stage and cause damping-off of seedlings, particularly when grown in the presence of nematodes whose damage from feeding may allow for the ingress of the pathogen [20,21]. Symptoms may vary (variable wilting, chlorosis, necrosis, and damping off), complicating the diagnosis of the disease. Variation is due to a number of factors such as environmental conditions, the concentration of pathogenic propagules, the race of the pathogen, age of the plant, and watermelon cultivar [1,14,22,23].

Although FON is a soilborne pathogen, it is internationally widespread with regions of historic watermelon production seeing the highest disease pressure [19,24,25]. Growers in the United States, particularly in Delaware, Florida, Georgia, Maryland, South Carolina, and Texas, continue to have difficulties controlling this disease with resistant cultivars [1,5,16,18,26,27,28]. Unfortunately, many previously resistant cultivars have become unsustainable as new populations of FON have emerged which can overcome the plant host resistance. These newly resistant populations have been designated as races that describe the unique interaction between pathogen and host [2,29,30]. While FON’s pathogenicity is limited to watermelon, four races of the pathogen are currently identified, race 0, race 1, race 2, and race 3, each subsequent race being more virulent with wider pathogenicity with race 3 having the largest cultivar range [1,4,29,30,31,32,33]. The first two races were described in 1963 when the newly pathogenic race 1 was discovered in Florida, dividing FON into races 0 and 1 [14]. Since that time, the other two races emerged in the United States: race 2 in 1981 in TX [14,34] and race 3 in 2009 in MD [1,14]. Race 2 is aggressive on all commercial watermelon cultivars but not the PI line (PI-296341-FR), and race 3 is aggressive on all watermelon cultivars and PI lines. Until recently, it was believed that race 1 was the most widespread race, race 2 less well distributed, and race 3 limited to a very small geographic area. As of today, race 3 has been detected in 3 states: MD [1], FL [31], and GA [32]. Recent survey studies in SC, GA, and FL have shown race 2 to be widespread, and in the case of GA, race 3 is also widespread, demonstrating the previously unknown dissemination of these highly aggressive races of FON [18,32]. 

Molecular detection methods have been developed for the differentiation of FON from other closely related formae speciales in the *F. oxysporum* species complex, as traditional methods of morphological identification cannot identify the pathogen beyond the species level [5,7,14,19,25]. Race differentiation, however, is limited to a bioassay, which is a lengthy, expensive, and inaccurate method. Seedlings of multiple cultivars with different levels of susceptibility are grown and inoculated with the pathogen and disease development is then scored. Based on the average score, the pathogen is given a race determination. This method takes weeks to grow plants and evaluate disease, the seeds of certain cultivars are difficult to source, and the experimental and greenhouse conditions (e.g., temperature, humidity, spore concentration, etc.) can have a significant effect on the level of pathogen virulence leading to an incorrect result [1,5,14,35,36]. In addition, a single race may have isolates that have vastly differing virulence on a single cultivar, not only due to the aforementioned experimental conditions but also possibly due to other unknown molecular mechanisms. Multiple isolates of race 1 can infect susceptible cultivars at disease levels ranging from 5 to 100% [37]. Thus, races are difficult to determine correctly based on phenotypic observations. These shortcomings demonstrate the necessity for a molecular approach for differentiation of FON races.

Niu et al. (2016) reported that on chromosome 14 of FON (also known as the pathogenicity chromosome), race 2 isolates lack the avirulence gene known as “*secreted in xylem protein 6*” (*SIX6*). By using polymerase chain reaction (PCR) primers specific to *SIX6*, race 2 could be differentiated molecularly from races 0 and 1. Race 3 isolates were not tested by Niu et al., however, upon whole-genome analysis, *AVRSIX6* was identified in the race 3 isolate, showing that the *AVRSIX6* primer could differentiate race 2 isolates from all other currently recognized races. 

The primary objective of this study was to develop a molecular method of differentiating FON race 3 isolates from race 1 and race 2 isolates to identify this new aggressive race rapidly and accurately. A comparison of the pathogenicity chromosome from published whole-genome sequences by Hudson et al. (2020) was used to find conserved and unique regions that could be used to design markers for race 3 specific amplification [38]. In addition, a FON race-differentiation protocol was developed by using the new PCR-based race 3 marker along with two other previously published FON-specific PCR markers, which was validated in race identification of 161 FON isolates collected from Florida, Georgia, and South Carolina.

## 2. Results

### 2.1. Sequence Analysis and Primer Design

**Figure 1:**
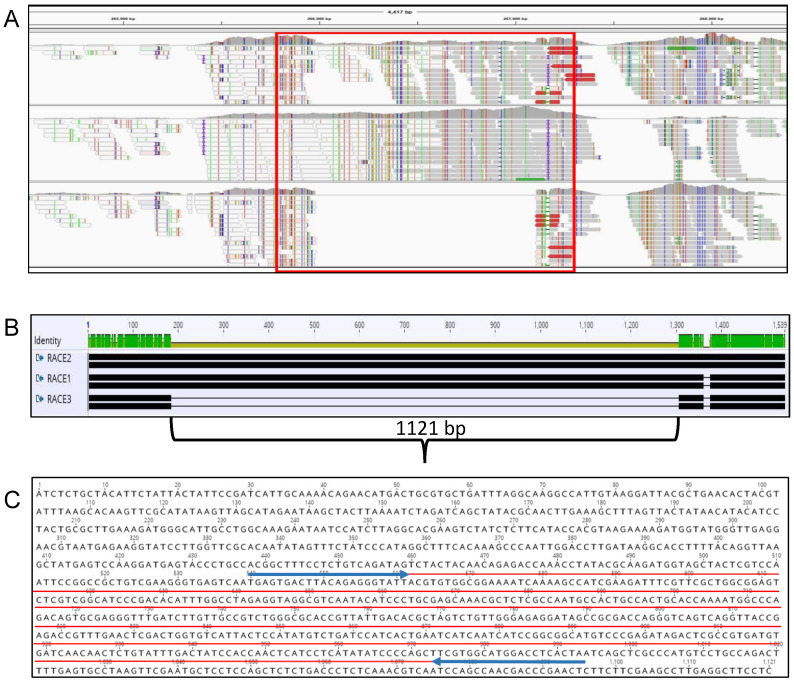
Region targeted for Polymerase Chain Reaction (PCR) amplification on chromosome 14 of *Fusarium oxysporum* f. sp. *niveum* (FON). (**A**) Interactive Genomics Viewer (IGV) window of races 1, 2, and 3. Red box indicates the area investigated for amplification. (**B**) Geneious alignment of races 1, 2, and 3, zoomed in on a 1540 bp section showing 1121-bp absent in race 3. (**C**) 1121 bp sequence on Geneious. Amplified sequence is underlined in red, forward and reverse primers are underlined in blue.

Genomic analysis of the three FON races used in this research revealed that the pathogenicity chromosome yielded several regions that were absent in race 3 and present in races 1 and 2. After testing the race standards in a larger pool of isolates, a single primer set was chosen (FNR3-F/FNR3-R) that amplified a 511-bp region of a larger region (1121 bp) absent in race 3 (Figure 1). This region also was chosen because of BLAST results identifying high homology (>90% identity) with a hypothetical protein from a *F. oxysporum* f. sp. *lycopersici* sequence (accession: XM_018387901), a *F. odoratissimum* (formerly *F. oxysporum* f. sp. *cubense*) sequence (accession: XM_031202915), and an unnamed *F. oxysporum* isolate from Australia (accession CP053262) [39,40]. Within the 1121-bp region absent in race 3 isolates, two separate open reading frames (ORFs) consisting of 74 and 62 amino acids are present as predicted by ORFfinder from the National Center for Biotechnology Information (NCBI) and the Interactive Genomic viewer (IGV).

### 2.2. Specificity and Sensitivity of the Race 3 Marker 

**Figure 2:**
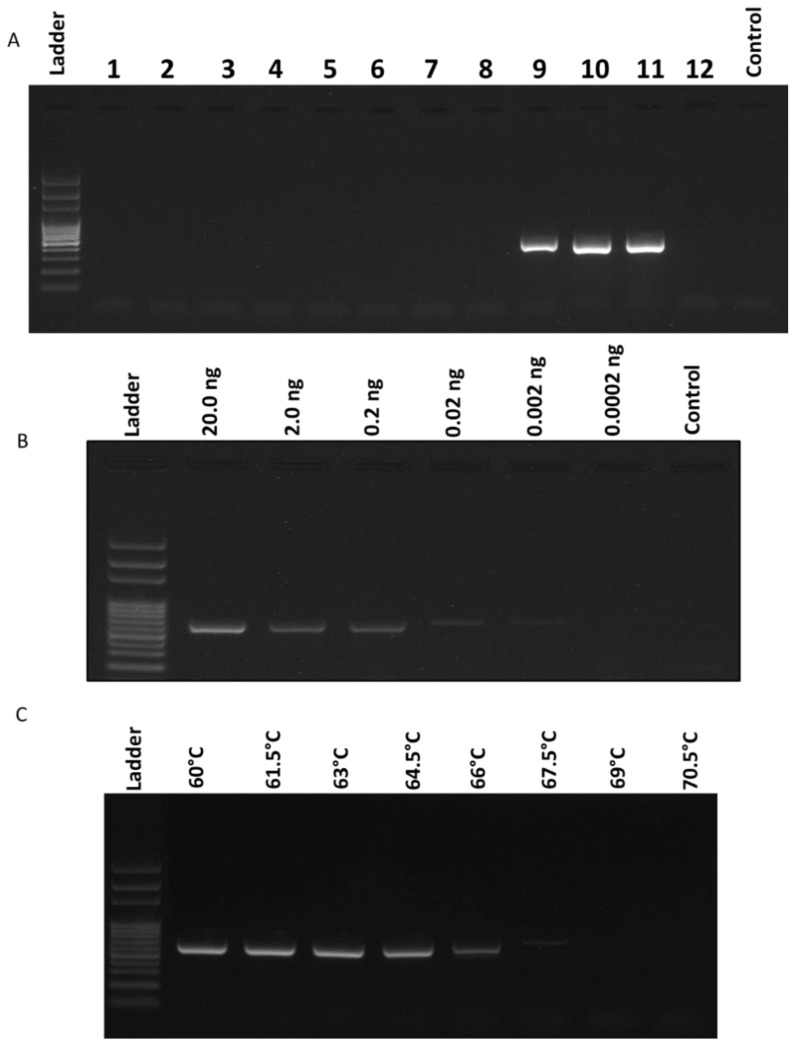
Optimization of the developed marker. (**A**) Non-target amplification of FNR3-F/FNR3-R. Sample numbers correspond accordingly: 1. *Phytophthora capsici*, 2. *Phytophthora sojae*, 3. *Pseudoperonospora cubensis*, 4. *Cucurbit leaf crumple virus*, 5. *Rhizoctonia solani*, 6. *Colletotrichum orbiculare*, 7. *Fusarium solani*, 8. *F. oxysporum* f. sp. *vasinfectum*, 9. *F. oxysporum* f. sp. *lycopersici*, 10. *Fusarium oxysporum* f. sp. *niveum* (FON) race 1, 11. FON race 2, 12. FON race 3, and control = negative. (**B**) Sensitivity determination of FNR3-F/FNR3-R using a serial dilution of FON DNA. DNA concentrations ranged from 20 ng/µL to 0.2 pg/µL. PCR positive bands amplified samples as low as 2.0 pg/µL. (**C**) Gradient PCR results to optimize annealing temperature. Temperatures ranged from 60 to 70.5 °C. The optimized temperature was 63 °C.

The race 3-specific marker, FNR3-F/FNR3-R, was optimized and tested for specificity and sensitivity (Figure 2). The specificity of the new marker was determined by testing additional cucurbit pathogens as well as other fungal and oomycete isolates: *Phytophthora capsici*, *Phytophthora sojae*, *Pseudoperonospora cubensis*, *Cucurbit leaf crumple virus*, *Rhizoctonia solani*, *Colletotrichum orbiculare*, *Fusarium solani*, *F. oxysporum* f. sp. *vasinfectum*, and *F. oxysporum* f. sp. *lycopersici*. 

Besides FON races 1 and 2, only *F. oxysporum* f. sp. *lycopersici* (FOL) was amplified using the same primer set and conditions. However, a slightly larger band size (555 bp) was amplified in FOL due to the differences in the individual base pairs of the FOL vs. FON amplicons (Figure 2A). To determine the sensitivity of the new marker, a serial dilution of the race 1 standard isolate was made from 20 ng/μL to 0.02 pg/μL and tested using the same PCR conditions. PCR results demonstrated the lowest successful amplification occurring at 2.0 pg/μL of genomic DNA (Figure 2B). The annealing temperature was determined to be optimized at 63 °C, showing the highest temperature with equally bright amplicons as lower temperatures (Figure 2C).

### 2.3. Development of a Protocol for Race Differentiation

**Figure 3:**
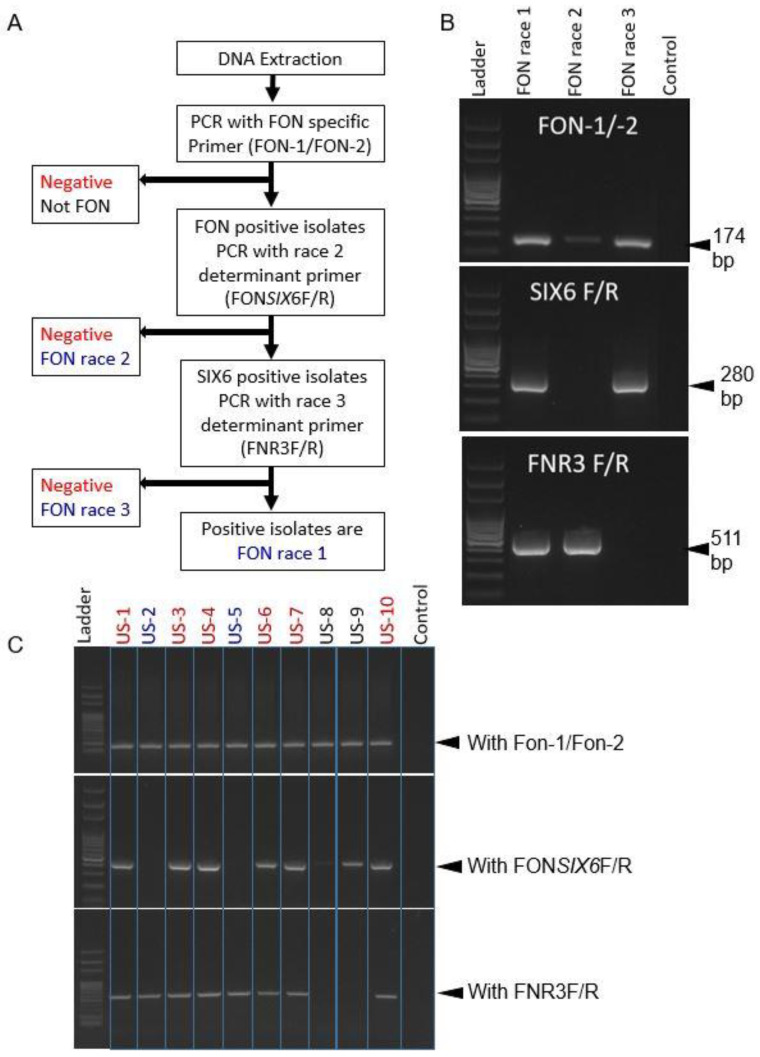
(**A**) Flowchart of *Fusarium oxysporum* f. sp. *niveum* (FON) isolate testing with all three marker sets to differentiate races 1, 2, and 3. Possible results are highlighted in blue. (**B**) Example of a practical application on known isolates. (**C**) Application on unknown field isolates. Absence of amplification of both FON*SIX6*F/R and FNR3-F/FNR3-R showed a positive identity for races 2 and 3.

A protocol was developed by using the new marker FNR3-F/FNR3-R in concert with the previously published Fon-1/Fon-2 and FON*SIX*6F/FON*SIX*6R markers, and differentiation of races 1, 2, and 3 from each other is possible by running them subsequently, as shown in Figure 3A. After DNA is isolated, samples are first confirmed with Fon-1/Fon-2 primers for their identity as FON isolates. Primer set FON*SIX6*F/FON*SIX6*R is then run to determine if the isolate is race 2 based on the absence of the amplicon. If there is an amplicon, samples are then amplified using FNR3-F/FNR3-R primers to determine if the isolate is race 1 or race 3, based on the absence (race 3) or presence (race 1) of the amplicon. This process is demonstrated in Figure 3C with 10 isolates of unknown identity to show what possible reactions can occur. Based on the results of these 10 isolates, two were identified as race 2 (US-2, 5) shown by a positive reaction with Fon-1/Fon-2 and FNR3-F/FNR3-R, but a negative reaction for FON*SIX*6F/R; two were identified as race 3 (US-8, 9) shown by a positive reaction for Fon-1/Fon-2, positive for FON*SIX*6F/R, and negative for FNR3-F/FNR3-R, and six were identified as race 1 (US-1, 3, 4, 6, 7, 10) as all reactions were positive.

### 2.4. Race Distribution of Experimental Samples

Two hundred one FON isolates from various field locations and labs in three states in the southeastern USA (FL, GA, and SC) were tested using the newly developed race differentiation protocol to establish their identity. FON samples that showed signs of contamination of any marker based on the gel images were removed or re-extracted and retested. All isolates were run with all markers in triplicate to confirm results. 

**Figure 4:**
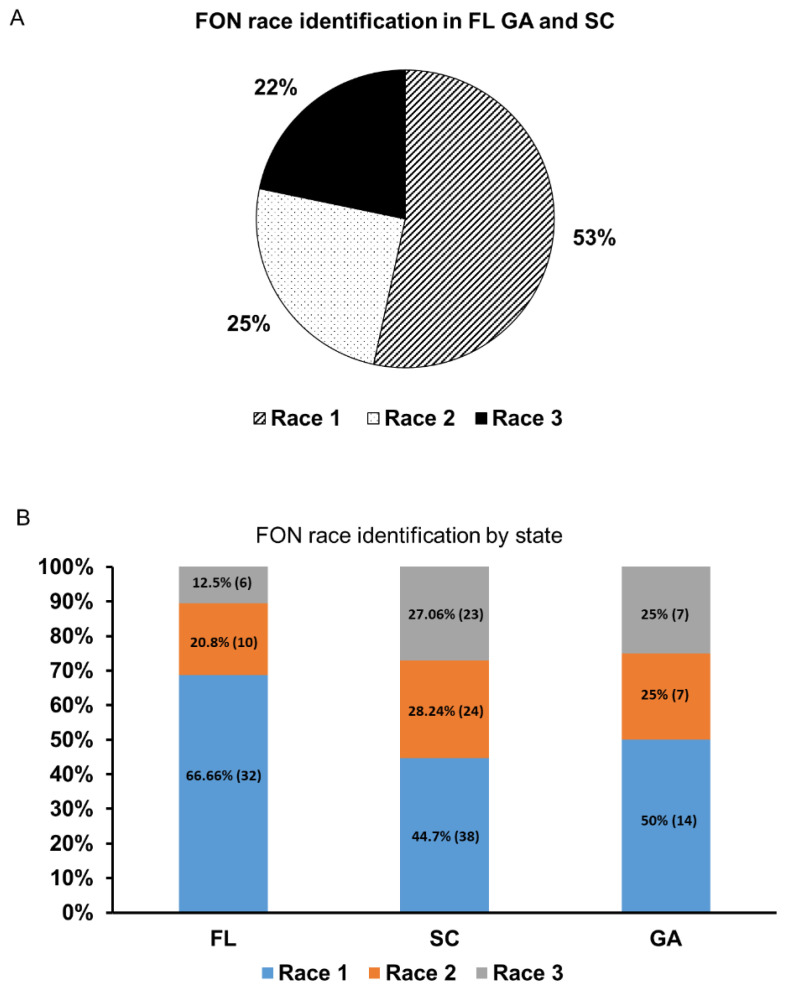
Molecular test results for race identification in states from which samples were taken. (**A**) Race distribution of all states tested. (**B**) Race distribution of individual state samples (FL: Florida, SC: South Carolina, and GA: Georgia). Race 1 = blue, race 2 = orange, race 3 = grey. The number of each race is labeled in the colored section corresponding to the race and state.

The method of determining race 1 isolates came only after using all the primer sets, as only race 1 isolates would show positive amplification from all three primer sets, whereas the other two races would be determined by their absence in amplification for each respective marker. After the removal of race 0 isolates identified by bioassays and non-FON samples (negative for Fon-1/Fon-2), 161 isolates remained and are presented with their PCR results and resulting race determination (Supplementary Table S1). The race distribution of all isolates tested, regardless of state was: 53% race 1, 25% race 2, and 22% race 3. Of the 28 GA isolates, 50% (14) were race 1, 25% (7) were race 2, and 25% (7) were race 3. Of the 85 SC isolates, 44.7% (38) were race 1, 28.24% (24) were race 2, and 27.06% (23) were race 3. Of the 48 FL isolates, 66.6% (32) were race 1, 20.8% (10) were race 2, and 12.5% (6) were race 3 (Figure 4). Additionally, isolates race-typed using the bioassay were compared to molecular assay results. Of those isolates, 26 (89.65%) for race 1 matched between the assays, 33 (80.49%) for race 2, and 14 (60.87%) for race 3 (Table 1). 

## 3. Discussion

The concept of races in various pathosystems is established, however, the requirement of a specific (a)virulence gene being identified as necessary for pathogenicity in order to confirm a race has been a recent addition [41,42,43]. This confirmation-by-correlation of *AVR* gene(s) to race has not been established in *Fusarium oxysporum* f. sp. *niveum*, as no genes have been confirmed to confer pathogenicity by being transferred to a less-virulent strain via horizontal chromosome transfer, so a more general approach is needed for race identification. Due to this, the bioassay that is used for FON race differentiation has a number of problems. First, experimental conditions (temperature, humidity, soil type, etc.) of the bioassay are difficult to standardize from lab to lab, not to mention from season to season. Second, isolates of a given race can change in virulence over time (perhaps due to other conditions such as long-term storage) and the scoring method to determine race, of which there are multiple, relies on the virulence level of an isolate [44,45,46]. Third, the cultivars used have discrepancies in the literature and because seeds of some cultivars are difficult to source, others must be substituted. Some researchers claim a range of resistance among seven cultivars, others use only four or five cultivars with a distinct delineation between their reactions, and still others substitute certain cultivars for others possibly changing the level of resistance again [4,10,47,48]. To confirm these inaccuracies between methodologies, copies of a single isolate were sent to multiple labs with access to the bioassay and different race results were returned. The genetic variability within a single race, or from isolate to isolate, is a known complexity in the *Fusarium oxysporum* species complex, characterized well by the lack of consistency when testing isolates with the traditional bioassay. Factors such as mobile pathogenicity chromosomes and horizontal chromosome transfer have been seen in other formae speciales and shed some light as to why certain markers do not remain successful over time, or in distinct geographic locations. Horizontal chromosome transfer presents the largest problem when maintaining consistency in evaluating virulence of isolates, as both the transfer from one forma specialis to another and from one *Fusarium* species to another have been documented [40,49,50,51]. 

As traditional methods for identification within the *F. oxysporum* species complex are known to be inadequate, molecular methods are necessary for accurate identification. Races complicate the process of molecular differentiation due to the highly conserved genomic content that they share (often < 1%) [32,42,52,53,54]. This can be seen through sequencing of traditional molecular marker genes such as *internal transcribed spacer* (*ITS*), heat shock proteins (HSP), *β-tubulin*, *intergenic spacer* (*IGS*), and *Cytochrome c oxidase* (*COX*), all of which were determined to have 100% conservation across all races sequenced. In addition, *F. oxysporum* is known to have the ability to transfer genes and chromosomes horizontally, both genes related and unrelated to pathogenicity [49,55,56,57]. As a result, a comparative genomics approach was used to identify genetic regions that would allow for consistent differentiation of race 3 from the other two races of economic impact: 1 and 2, which are currently increasing in presence across the world [1,24,31,58,59,60]. No race 0 isolate was available for analysis or comparison. The whole-genome sequencing results were published previously by Hudson et al. (2020) which contain more analysis and details of the WGS data [38]. It was noted that during testing, race 0 isolates identified by the bioassay were most commonly negative for FON*SIX6*F/R, signifying race 2 as the identity, but other race 0 isolates had variable results including negative for both Fon-1/Fon-2 and FNR3 primer sets. Additional isolates tested in the bioassay were nonpathogenic on susceptible cultivars and pathogenic on the resistant PI line which did not allow for proper race differentiation (J. Fulton, personal communication). The variation of isolate pathogenicity and race 0 variability underlines the necessity for additional genes to be sequenced and correlated closely with pathogenicity.

In an attempt to neutralize some of the variations from the bioassay results, several steps were made to confirm that the genomic sequences of each race were accurate to the claimed race and the region in the genome chosen for marker development would be conserved. As the key importance between races is differential pathogenicity, and due to a high concentration of SNPs and InDels, we focused on chromosome 14, where previous studies on FON and other *F. oxysporum* formae speciales had identified avirulence genes [37,40,49,61,62]. Other chromosomes (3, 6, and 15) are additionally known to be involved in pathogenicity but were not used as fewer genetic changes were seen [14]. The primary group of these avirulence genes is the “secreted in xylem” or *SIX* genes [57,63,64]. One such gene, *SIX6*, was developed previously and used in this study to differentiate race 2 isolates based on the absence of *SIX6* [3,65,66]. The identity of the race 2 whole genome sequence was additionally confirmed through chromosomal analysis to lack the *SIX6* gene region, implying that the race 2 isolate was consistent with previous research. This method of genomic confirmation gave better confidence to molecular results as they correlate with race differentiation; both race 1 and race 3 have identical copies of *SIX6*, also suggesting separate origins for races 2 and 3.

In this study, the FNR3 marker was developed using comparative genomics in an attempt to provide stable locations in the FON genome that will rapidly determine a FON isolate as a highly virulent one, currently characterized as race 3. This marker was designed based on a unique region on the “pathogenicity chromosome” of the FON genome, absent in race 3 but present in races 1 and 2. This marker was found to be effective to differentiate race 3 from other races and no false negatives or false positives were observed during the validation with other phytopathogens except *F. oxysporum* f. sp. *lycopersici* (FOL) (Figure 2). The sensitivity of this assay revealed the detection limit of the primer set to be 2.0 pg/μL and the optimized annealing temperature was 63 °C for 30 s (Figure 2B,C). In order to determine the race (1, 2, or 3) of an isolate that is pathogenic on the least resistant cultivar, a protocol was developed that outlines all possible results from testing a FON isolate with the three primer sets (Figure 3A). Multiplex PCR was attempted for this study, but consistent results could not be obtained. It was probably because cycle conditions differed too greatly, or inhibition due to multiple PCR primers occurred. It is important to note that race 1 isolates must receive a positive reaction from all three primer sets to confirm race 1 as the identity, but the initial Fon-1/Fon-2 primer set is required for all FON identifications (Figure 3). 

After race typing, isolates were rearranged to reflect their geographic state of origin and assessed on that basis. In this study, the races of available isolates we identified were as follows for all three states combined: race 1: 53.4%, race 2: 24.84%, and race 3: 21.74% (Figure 4). Of the three states, only two had previously reported the presence of race 3 FON isolates (Florida and Georgia) but South Carolina had not [1,18,31,32]. According to the molecular test results, this would be the first time race 3 has been detected in SC, however, previous studies have addressed the lack of a race 3 phenotype in SC based on testing of a number of cultigens of watermelon. While isolates and locations were not sampled randomly as in a survey, GA isolates differed in percentages from previously reported studies in which race 3 had been the most common race, instead of race 1 in this study [32]. The percent correlation (%) of race differentiation between bioassay vs. molecular assay was determined based on a sample of 93 isolates that were tested for race determination by the two methods (Supplementary Table S2). The percentage (%) of molecular results agree with the bioassay results of race 1, 2, and 3 at 89.65%, 80.49%, and 60.87%, respectively (Table 1). A significant disagreement was observed between the two approaches for race identification, which may be due to the aforementioned experimental variation within the bioassay, long-term storage of some isolates, or inconsistencies with cultivar usage and reaction with the pathogen. Alternatively, race 3 isolates, as they were only recently recognized within the literature, could be a group of other, yet uncharacterized, races all with higher virulence than race 2 isolates. The additional possibility, specifically to *Fusarium* spp., of horizontal chromosome shifting would theoretically allow for variability of a single isolate, causing alterations in results of both bioassay and molecular assay. 

Based on previously published reports on pathogenic races, it is hypothesized that due to loss of function or absence of avirulence (*AVR*) genes in specific races, the pathogen could circumvent the resistance of the plant host [3,67,68,69]. While the targeted region for race 3 differentiation is not a known *AVR* gene, similar processes of novel resistance could be occurring. This would be evidence against the thesis of sequential development of races in the order in which they have been detected but instead races 2 and 3 arising from a common origin such as race 0 or race 1. Alternatively, gene acquisition conferring quantitative disease resistance could play a significant role in the development of newly pathogenic races, as has been seen in other *Fusarium* spp. Further analysis of international FON samples of all races is necessary to increase the confidence of marker stability and determine the mechanisms of resistance. This means that the availability of more whole-genome sequences like the ones used in this study will allow easier marker design and comparison in the future. Specifically, multiple genomes of isolates that are at the extremes of virulence should be analyzed and compared as should isolates from distinct geographic origins. In addition to the region selected for amplification with the FNR3-F/FNR3-R primer set, screening of possible effector protein-coding regions in the FON genome would be a reasonable next step for the development of knockout mutants to test pathogenicity on resistant watermelon cultivars and to connect the identity of a specific FON race to an *AVR* gene. The marker presented in this study should improve the speed and accuracy of the current diagnostic ability for FON and provide a jumping off point for other researchers to investigate similar regions involved in pathogenicity and race development. 

## 4. Materials and Methods

### 4.1. FON Isolates and DNA Extraction

FON isolates were gathered from laboratories of Ji, Dufault, and Keinath at the University of Georgia, University of Florida, and Clemson University, respectively (Supplementary Table S1). Many of the isolates were previously identified to races using greenhouse bioassay by the respective labs. No bioassay testing was done in this study. The isolates were grown on potato dextrose agar (PDA) plates at 25 °C for seven days, and fungal tissue (100 mg) was scraped into a 1.5-mL safe lock tube (Eppendorf Canada Ltd., Mississauga, ON, Canada) with steel beads for homogenization. Samples were homogenized in the FastPrep FP120 cell disruptor (Qbiogene, Carlsbad, CA, USA) for 30 s at speed 5, twice, or until there were no large pieces of mycelia. DNA was extracted using the DNeasy plant mini kit (Qiagen, Valencia, CA, USA) according to the manufacturer’s instructions. DNA was purified using Quantum Prep PCR Kleen Spin Columns (BIO-RAD, Hercules, CA, USA). Total DNA yield and purity were estimated by measuring OD at 260 nm and 260/280 nm with a NanoDrop LITE (Thermo Scientific, Waltham, MA, USA).

### 4.2. Sequence Alignment and Primer Design

Genomic sequences of FON isolates (BioProject number: PRJNA656528 and accession numbers SAMN15791673, SAMN15791674, and SAMN15791675) and the reference genome of FOL 4287 (NCBI: txid426428) were obtained from NCBI [38,49]. Sequences were aligned and visualized using the Interactive Genomics Viewer (IGV) (2013-2018 Broad institute and the Regents of the University of California). Targets for primer design were searched on chromosome 14 (NC_030999.1), as it contained previously targeted gene candidates for race differentiation [40]. Regions absent in race 3 but present in races 1 and 2 were targeted specifically [40]. Using this approach, 21 different loci were isolated as candidates for differentiation based on their absence in the race 3 genome. To narrow the possible pool of target loci, BLAST was used to determine the likelihood of genetic conservation within the genome based on shared sequences with related species. In addition, sequences containing coding regions and hypothetical proteins were targeted specifically for primer design. After using BLAST, a further narrowing of candidates was done based on primer design properties such as GC content analysis and amplicon size. Consequently, seven primer pairs were then designed manually and checked for quality and content using the Integrated DNA Technologies PrimerQuest Tool. All primers were synthesized by Sigma Aldrich (St. Louis, MO, USA) and stored at −20 °C. All primers are listed in Table 2.

### 4.3. Selection of Race-Specific Diagnostic Marker Primer Set and PCR Conditions

To select a marker that specifically amplified races 1 and 2 but not race 3, all primer sets were tested against the three isolates sequenced by Hudson et al. (2020), then on a larger pool of isolates from multiple states (20 from each state) [38]. Primers with weak signals, strong dimers, or double bands were removed. Finally, two primer sets remained and were used to amplify all FON isolate DNA. From these PCR tests, a final primer set was chosen after showing clear and consistent amplification of target isolates. The final primer set was a result of targeting an 1121-bp region in chromosome 14 that was identical in races 1 and 2, and entirely absent in race 3 (Figure 1). The primer, named FNR3-F/FNR3-R, amplified a 511 bp (555 bp in the FOL reference) region of the larger 1121-bp region that contained multiple hypothetical proteins depending on the codon frame viewed. PCR reactions were executed on a thermal cycler (Biorad-96 well T100™, Bio-rad, Hercules, CA, USA) using EconoTaq^®^ PLUS Green 2X Master Mix (Lucigen, Middleton, WI, USA) which included the following components: EconoTaq^®^ PLUS Green 2X Master Mix (12.5 µL), forward primer (0.3 µM), reverse primer (0.3 µM), target DNA (1 µL), and ddH2O to total 25 µL per reaction. PCR products (4 μL per sample) were run on a 1% agarose gel stained with SYBR safe stain (Invitrogen, Waltham, MA, USA) and then imaged on a UV gel doc (Analytik Jena, Jena, Germany). PCR was performed using the following conditions: denaturation at 95 °C for 3 min followed by 35 cycles of amplification at 95 °C for 30 s, annealing at 63 °C for 30 s and 72 °C for 40 s, and terminated by a final elongation at 72 °C for 6 min.

### 4.4. Optimization of the Developed Marker

The new primer set was tested to determine the optimal annealing temperature, the level of specificity, and the detection limit of genomic DNA. To optimize the annealing temperature a gradient PCR was run from 60 to 70.5 °C and the highest temperature was selected that did not diminish the brightness of the amplicon. To analyze the specificity, the FNR3-F/FNR3-R primer set was tested with a range of non-FON isolates. PCR samples were made to the same concentrations as described above with approximately 50 ng of DNA per reaction. A positive FON control and a negative water control were included with the non-target samples. Other pathogens tested were as follows: *Phytophthora capsici*, *Phytophthora sojae*, *Pseudoperonospora cubensis*, *Cucurbit leaf crumple virus*, *Rhizoctonia solani*, *Colletotrichum orbiculare*, *Fusarium solani*, *F. oxysporum* f. sp. *vasinfectum*, and *F. oxysporum* f. sp. *lycopersici*. To test the sensitivity levels of the newly designed PCR marker, the genomic DNA extracted from the race 1 standard isolate was standardized to 20 ng/μL, then underwent a tenfold serial dilution down to 0.2 pg/μL of DNA. These dilutions were amplified using the new primer set FNR3-F/FNR3-R to determine the minimum sensitivity. PCR results (4 μL per sample) were imaged on a 1% agarose gel and the lowest successful amplification was determined to be the detection limit of the new primer set. 

### 4.5. Development and Application of a Protocol for Race Differentiation

A protocol with three PCR primer sets was used to amplify and determine races of the FON isolates (Figure 1). The first primer set (Fon-1/Fon-2) was used to amplify FON isolates specifically, with no other pathogenic *F. oxysporum* formae speciales being amplified [19]. The second primer set (FON*SIX6*-F/ FON*SIX6*-R) was used to determine race 2 isolates based on the absence of the *SIX6* gene [3]. The final primer set “FNR3-F/FNR3-R” was developed in this study and selectively amplifies races 1 and 2 and has no amplicon for race 3 isolates. Two hundred one FON isolates from various field locations and labs in Florida, Georgia, and South Carolina were tested using the new protocol with the three primer sets to establish their identity: Fon-1/Fon-2 to confirm their identity as FON, FON*SIX6*F/FON*SIX6*R to differentiate race 2 from races 1 and 3, and FNR3-F/FNR3-R to differentiate race 3 isolates.

## Figures and Tables

**Table 1:** Percentage of agreement for race differentiation using bioassay vs. molecular assay.

FON Race	Number of Isolates Tested Using Both Methods	Percentage (%) of Molecular Results Agree with the Bioassay Race Results
Race 1	29	89.65%
Race 2	41	80.49%
Race 3	23	60.87%

**Table 2:** List of primers used in this study.

Assay	Primers	Sequence (5′–3′)	Product Size (bp)	Source
FON specific primer	Fon-1	CGATTAGCGAAGACATTCACAAGACT	174	[19]
	Fon-2	ACGGTCAAGAAGATGCAGGGTAAAGGT		
Race 2 differentiating primer	FON*SIX6*F	CGCTCTTATCGCATCAATCT	453	[3]
	FON*SIX6*R	GGGTTGACTGAGGTCGTGGT		
Race 3 differentiating primer	FNR3F	CGGCTTTCCTCTGTCAGATAGT	511	This study
	FNR3R	TAGTGAGGTCCATGCCACGAA		

## Data Availability

Data available in a publicly accessible repository that does not issue DOIs. Publicly available datasets were analyzed in this study. This data can be found here: Accession numbers: SAMN15791673, SAMN15791674, and SAMN15791675.

## Supplementary Materials

Supplementary materials can be found at https://www.mdpi.com/1422-0067/22/2/822/s1.

## Abbreviations

FON	*Fusarium oxysporum* f. sp. *niveum*
SIX6	Secreted in Xylem 6
PDA	Potato Dextrose Agar
PCR	Polymerase Chain Reaction
BLAST	Basic Local Alignment Search Tool
FOL	*Fusarium oxysporum* f. sp. *lycopersici*
IGV	Interactive Genomics Viewer
NCBI	National Center for Biotechnology Information
HSP	Heat Shock Protein
ITS	Internal Transcribed Spacer
COX	Cytochrome c Oxidase
IGS	Intergenic Spacer
AVR	Avirulence Gene
PI	Plant Introduction

## References

[1] Zhou X., Everts K., Bruton B. (2010). Race 3, a new and highly virulent race of Fusarium oxysporum f. sp. niveum causing Fusarium wilt in watermelon. Plant Dis..

[2] Netzer D., Weintall C. (1980). Inheritance of resistance in watermelon to race 1 of Fusarium oxysporum f. sp. niveum. Plant Dis..

[3] Niu X., Zhao X., Ling K.-S., Levi A., Sun Y., Fan M. (2016). The FonSIX6 gene acts as an avirulence effector in the Fusarium oxysporum f. sp. niveum-watermelon pathosystem. Sci. Rep..

[4] Roberts P., Dufault N., Hochmuth R., Vallad G., Paret M. (2019). Fusarium Wilt (Fusarium oxysporum f. sp. niveum) of Watermelon.

[5] Egel D., Martyn R. (2007). Fusarium wilt of watermelon and other cucurbits. Plant Health Instr..

[6] Edel-Hermann V., Lecomte C. (2019). Current status of Fusarium oxysporum formae speciales and races. Phytopathology.

[7] Peng J., Zhan Y., Zeng F., Long H., Pei Y., Guo J. (2013). Development of a real-time fluorescence loop-mediated isothermal amplification assay for rapid and quantitative detection of Fusarium oxysporum f. sp. niveum in soil. FEMS Microbiol. Lett..

[8] Akhter A., Hage-Ahmed K., Soja G., Steinkellner S. (2016). Potential of Fusarium wilt-inducing chlamydospores, in vitro behaviour in root exudates and physiology of tomato in biochar and compost amended soil. Plant Soil.

[9] McKeen C., Wensley R. (1961). Longevity of Fusarium oxysporum in soil tube culture. Science.

[10] Kleczewski N.M., Egel D.S. (2011). A diagnostic guide for Fusarium wilt of watermelon. Plant Health Prog..

[11] Chittarath K., Mostert D., Crew K.S., Viljoen A., Kong G., Molina A., Thomas J.E. (2018). First report of Fusarium oxysporum f. sp. cubense tropical race 4 (VCG 01213/16) associated with Cavendish bananas in Laos. Plant Dis..

[12] Manulis S., Kogan N., Reuven M., Ben-Yephet Y. (1994). Use of the RAPD technique for identification of Fusarium oxysporum f. sp. dianthi from carnation. Phytopathology.

[13] Quesada-Ocampo L. (2018). Fusarium Wilt of Watermelon.

[14] Martyn R.D. (2014). Fusarium wilt of watermelon: 120 years of research. Hortic. Rev..

[15] Hutson R., Smith I. (1980). Phytoalexins and tyloses in tomato cultivars infected with Fusarium oxysporum f. sp. lycopersici or Verticillium albo-atrum. Physiol. Plant Pathol..

[16] Bruton B., Fish W., Langston D. (2008). First report of Fusarium wilt caused by Fusarium oxysporum f. sp. niveum race 2 in Georgia watermelons. Plant Dis..

[17] Zhang M., Xu J., Liu G., Yao X., Li P., Yang X. (2015). Characterization of the watermelon seedling infection process by Fusarium oxysporum f. sp. niveum. Plant Pathol..

[18] Keinath A.P., DuBose V., Katawczik M., Wechter P. (2020). Identifying races of Fusarium oxysporum f. sp. niveum in South. Carolina Recovered from Watermelon Seedlings, Plants, and Field Soil. Plant Dis..

[19] Lin Y.-H., Chen K.-S., Chang J.-Y., Wan Y.-L., Hsu C.-C., Huang J.-W., Chang P.-F.L. (2010). Development of the molecular methods for rapid detection and differentiation of Fusarium oxysporum and F. oxysporum f. sp. niveum in Taiwan. New Biotechnol..

[20] Sumner D.R., Johnson A.W. (1973). Effect of root-knot nematodes on Fusarium wilt of watermelon. Phytopathology.

[21] Hua G.K.H., Timper P., Ji P. (2019). Meloidogyne incognita intensifies the severity of Fusarium wilt on watermelon caused by Fusarium oxysporum f. sp. niveum. Can. J. Plant Pathol..

[22] Keinath A.P., Coolong T.W., Lanier J.D., Ji P.J.P.D. (2019). Managing Fusarium wilt of watermelon with delayed transplanting and cultivar resistance. Plant Dis..

[23] Okungbowa F., Shittu H. (2012). Fusarium wilts: An overview. Environ. Res. J..

[24] Kurt S., Dervis S., Soylu E.M., Tok F.M., Yetisir H., Soylu S. (2008). Pathogenic races and inoculum density of Fusarium oxysporum f. sp. niveum in commercial watermelon fields in southern Turkey. Phytoparasitica.

[25] Zhang Z., Zhang J., Wang Y., Zheng X. (2005). Molecular detection of Fusarium oxysporum f. sp. niveum and Mycosphaerella melonis in infected plant tissues and soil. FEMS Microbiol. Lett..

[26] Everts K., Hochmuth M. (2011). Field evaluation of triploid cultivars for resistance to Fusarium wilt of watermelon in Delaware, 2010. Plant Dis. Manag. Rep..

[27] Everts K.L., Himmelstein J.C. (2015). Fusarium wilt of watermelon: Towards sustainable management of a re-emerging plant disease. Crop Prot..

[28] Bruton B., Damicone J. (1999). Fusarium wilt of watermelon: Impact of race 2 of Fusarium oxysporum f. sp. niveum on watermelon production in Texas and Oklahoma. Subtrop. Plant Sci..

[29] Wechter W.P., Kousik C., McMillan M., Levi A. (2012). Identification of resistance to Fusarium oxysporum f. sp. niveum race 2 in Citrullus lanatus var. citroides plant introductions. HortScience.

[30] Keinath A.P., Hassell R.L., Everts K.L., Zhou X.-G. (2010). Cover crops of hybrid common vetch reduce Fusarium wilt of seedless watermelon in the eastern United States. Plant Health Prog..

[31] Amaradasa B., Beckham K., Dufault N., Sanchez T., Ertek T., Iriarte F., Paret M., Ji P. (2018). First report of Fusarium oxysporum f. sp. niveum race 3 causing wilt of watermelon in Florida, USA. Plant Dis..

[32] Petkar A., Harris-Shultz K., Wang H., Brewer M.T., Sumabat L., Ji P. (2019). Genetic and phenotypic diversity of Fusarium oxysporum f. sp. niveum populations from watermelon in the southeastern United States. PLoS ONE.

[33] Liu X., Lv H., Yang Y., Xing M.M., Kong C.C., Fang Z., Yang L., Zhang Y., Wang Y., Ling J. (2019). Genetic diversity, virulence, race profiling and comparative genomic analysis of the Fusarium oxysporum f. sp. conglutinans strains infecting cabbages in China. Front. Microbiol..

[34] Martyn R. (1985). An aggressive race of Fusarium oxysporum f. sp. niveum new to the United States. Plant Dis..

[35] Martyn R. (1989). An initial survey of the United States for races of Fursarium oxysporum f. sp. niveum. HortScience.

[36] Larkin R., Hopkins D., Martin F. (1990). Vegetative compatibility within Fusarium oxysporum f. sp. niveum and its relationship to virulence, aggressiveness, and race. Can. J. Microbiol..

[37] Fulton C.J., Amaradasa B.S., Ertek S.T., Iriarte F., Sanchez T., Ji P., Paret M., Hudson O., Ali E., Dufault S.N. (2020). Phylogenetic and phenotypic characterization of Fusarium oxysporum f. sp. niveum isolates from Florida-grown watermelon. PLoS ONE.

[38] Hudson O., Hudson D., Ji P., Ali M.E. (2020). Draft genome sequences of three Fusarium oxysporum f. sp. niveum isolates used in designing markers for race differentiation. Microbiol. Resour. Announc..

[39] Larkin R., Hopkins D., Martin F. (2020). A chromosome-scale genome assembly for the Fusarium oxysporum strain Fo5176 to establish a model Arabidopsis-fungal pathosystem. Genes Genomes Genet..

[40] Ma L.-J., Van Der Does H.C., Borkovich K.A., Coleman J.J., Daboussi M.-J., Di Pietro A., Dufresne M., Freitag M., Grabherr M., Henrissat B. (2010). Comparative genomics reveals mobile pathogenicity chromosomes in Fusarium. Nature.

[41] Inami K., Yoshioka-Akiyama C., Morita Y., Yamasaki M., Teraoka T., Arie T. (2012). A genetic mechanism for emergence of races in Fusarium oxysporum f. sp. lycopersici: Inactivation of avirulence gene AVR1 by transposon insertion. PLoS ONE.

[42] Kistler H. (1997). Genetic diversity in the plant-pathogenic fungus Fusarium oxysporum. Phytopathology.

[43] Mehmood S., Sajid M., Husnain S.K., Zhao J., Huang L., Kang Z. (2020). Study of inheritance and linkage of virulence genes in a selfing population of a Pakistani dominant race of Puccinia striiformis f. sp. tritici. Int. J. Mol. Sci..

[44] Porter L.D., Pasche J.S., Chen W., Harveson R.M. (2015). Isolation, identification, storage, pathogenicity tests, hosts, and geographic range of Fusarium solani f. sp. pisi causing fusarium root rot of pea. Plant Health Prog..

[45] Windels C.E., Burnes P.M., Kommedahl T. (1988). Five-year preservation of Fusarium species on silica gel and soil. Phytopathology.

[46] Webb K., Hill A., Laufman J., Hanson L., Panella L. (2011). Long-term preservation of a collection of Rhizoctonia solani using cryogenic storage. Ann. Appl. Biol..

[47] Zhou X., Everts K. (2001). First report of the occurrence of Fusarium oxysporum f. sp. niveum race 2 in commercial watermelon production areas of Maryland and Delaware. Plant Dis..

[48] Dutta B., Coolong T. (2017). Fusarium Wilt of Watermelon in Georgia.

[49] van Dam P., Fokkens L., Ayukawa Y., van der Gragt M., Ter Horst A., Brankovics B., Houterman P.M., Arie T., Rep M. (2017). A mobile pathogenicity chromosome in Fusarium oxysporum for infection of multiple cucurbit species. Sci. Rep..

[50] Vlaardingerbroek I., Beerens B., Rose L., Fokkens L., Cornelissen B.J., Rep M. (2016). Exchange of core chromosomes and horizontal transfer of lineage-specific chromosomes in Fusarium oxysporum. Environ. Microbiol..

[51] Mehrabi R., Bahkali A.H., Abd-Elsalam K.A., Moslem M., Ben M’Barek S., Gohari A.M., Jashni M.K., Stergiopoulos I., Kema G.H., de Wit P.J. (2011). Horizontal gene and chromosome transfer in plant pathogenic fungi affecting host range. FEMS Microbiol. Rev..

[52] Sakr N. (2013). Pathogenic, morphological and genetic diversity in Plasmopara halstedii, the causal agent of sunflower downy mildew. Acta Scientiarum. Agronomy.

[53] Martín-Sanz A., Rueda S., García-Carneros A.B., González-Fernández S., Miranda-Fuentes P., Castuera-Santacruz S., Molinero-Ruiz L. (2018). Genetics, host range, and molecular and pathogenic characterization of Verticillium dahliae from sunflower reveal two differentiated groups in Europe. Front. Plant Sci..

[54] Henrique F.H., Carbonell S.A.M., Ito M.F., Gonçalves J.G.R., Sasseron G.R., Chiorato A.F. (2015). Classification of physiological races of Fusarium oxysporum f. sp. phaseoli in common bean. Bragantia.

[55] Liu S., Wu B., Shen Z., Li R., Ganjun Y., Li C., Guo X. (2019). Genetic diversity in FUB genes of Fusarium oxysporum f. sp. cubense suggest horizontal gene transfer. Front. Plant Sci..

[56] Laurence M., Summerell B., Liew E. (2015). Fusarium oxysporum f. sp. canariensis: Evidence for horizontal gene transfer of putative pathogenicity genes. Plant Pathol..

[57] Czislowski E., Fraser-Smith S., Zander M., O’Neill W.T., Meldrum R.A., Tran-Nguyen L.T., Batley J., Aitken E.A. (2018). Investigation of the diversity of effector genes in the banana pathogen, Fusarium oxysporum f. sp. cubense, reveals evidence of horizontal gene transfer. Mol. Plant Pathol..

[58] Pal S., Rao E.S., Thontadarya R., Chandran N.K., Sriram S. (2020). First report on the occurrence of races 1 and 2 of Fusarium oxysporum f. sp. niveum infecting watermelon in India. Indian Phytopathol..

[59] Boughalleb N., El-Mahjoub M. (2005). Detection of races 0, 1 and 2 of Fusarium oxysporum f. sp. niveum and their distribution in the watermelon-growing regions of Tunisia. EPPO Bull..

[60] Boughalleb-M’Hamdi N., Salem I.B., BenFradj N., Abad-Campus P. (2020). Genetic diversity of Fusarium oxysporum f. sp. niveum responsible of watermelon Fusarium wilt in Tunisia and Spain. J. Phytopathol. Pest Manag..

[61] Rep M., Kistler H.C. (2010). The genomic organization of plant pathogenicity in Fusarium species. Curr. Opin. Plant Biol..

[62] Ma L.-J., Geiser D.M., Proctor R.H., Rooney A.P., O’Donnell K., Trail F., Gardiner D.M., Manners J.M., Kazan K. (2013). Fusarium pathogenomics. Annu. Rev. Microbiol..

[63] Taylor A., Vágány V., Jackson A.C., Harrison R.J., Rainoni A., Clarkson J.P. (2016). Identification of pathogenicity-related genes in Fusarium oxysporum f. sp. cepae. Mol. Plant Pathol..

[64] Rep M., Meijer M., Houterman P., Van Der Does H., Cornelissen B. (2005). Fusarium oxysporum evades I-3-mediated resistance without altering the matching avirulence gene. Mol. Plant Microbe Interact..

[65] van Dam P., de Sain M., Ter Horst A., van der Gragt M., Rep M. (2018). Use of comparative genomics-based markers for discrimination of host specificity in Fusarium oxysporum. Appl. Environ. Microbiol..

[66] Gawehns F., Houterman P., Ichou F.A., Michielse C., Hijdra M., Cornelissen B., Rep M., Takken F. (2014). The Fusarium oxysporum effector Six6 contributes to virulence and suppresses I-2-mediated cell death. Mol. Plant Microbe Interact..

[67] Oelke L.M., Bosland P.W., Steiner R. (2003). Differentiation of race specific resistance to Phytophthora root rot and foliar blight in Capsicum annuum. J. Am. Soc. Hortic. Sci..

[68] Kashiwa T., Suzuki T., Sato A., Akai K., Teraoka T., Komatsu K., Arie T. (2016). A new biotype of Fusarium oxysporum f. sp. lycopersici race 2 emerged by a transposon-driven mutation of avirulence gene AVR1. FEMS Microbiol. Lett..

[69] Lievens B., Houterman P.M., Rep M. (2009). Effector gene screening allows unambiguous identification of Fusarium oxysporum f. sp. lycopersici races and discrimination from other formae speciales. FEMS Microbiol. Lett..

