# Supplementary material for: Marker Development for Differentiation of Fusarium oxysporum f. sp. Niveum Race 3 from Races 1 and 2
Source: Int J Mol Sci. 2021 Jan 15;22(2):822. doi: 10.3390/ijms22020822 (PMC7830397; doi:10.3390/ijms22020822)
Supplement: Supplementary file 1 [file ijms-22-00822-s001.pdf]

Supplementary materials

Marker Development for Differentiation of *Fusarium oxysporum* f. sp. *niveum* Race 3 from Races 1 and 2

Owen Hudson [1], Sumyya Waliullah [1], James C. Fulton [2], Pingsheng Ji [1], Nicholas Dufault [2], Anthony Keinath [3] and Md Emran Ali [1,*]

[1] Department of Plant Pathology, University of Georgia, Tifton, GE 31793, USA; Owen.hudson@uga.edu (O.H.); Sumyya.Waliullah@uga.edu (S.W.); pji@uga.edu (P.J.)
[2] Department of Plant Pathology, University of Florida, Gainesville, FL 32611, USA; pcvgt@ufl.edu (J.C.F.); Nsdufault@ufl.edu (N.D.)
[3] Department of Plant and Environmental Sciences, Clemson University, Charleston, SC 29414, USA; tknth@clemson.edu
* Correspondence: emran.ali@uga.edu

Supplemental Table S1. Race differentiation using PCR assays with all pathogenic FON isolates tested.

Sl. no	Isolate Name	Source	Tested primer			Race
			FON1-2	SIX6F/R	FNR3	
1	GA-1	GA	P	N	P	R2
2	GA-2	GA	P	N	P	R2
3	GA-3	GA	P	N	P	R2
4	GA-4	GA	P	P	N	R3
5	GA-5	GA	P	N	P	R2
6	GA-6	GA	P	P	N	R3
7	GA-7	GA	P	P	N	R3
8	GA-8	GA	P	P	P	R1
9	GA-9	GA	P	P	N	R3
10	GA-10	GA	P	P	P	R1
11	GA-11	GA	P	P	P	R1
12	GA-12	GA	P	N	P	R2
13	GA-13	GA	P	P	P	R1
14	GA-14	GA	P	P	P	R1
15	GA-15	GA	P	P	P	R1
16	GA-16	GA	P	P	P	R1
17	GA-17	GA	P	P	P	R1
18	GA-18	GA	P	P	P	R1
19	GA-19	GA	P	P	P	R1
20	GA-20	GA	P	P	N	R3
21	GA-21	GA	P	P	P	R1
22	GA-22	GA	P	P	P	R1
23	GA-23	GA	P	P	N	R3
24	GA-24	GA	P	P	N	R3
25	GA-25	GA	P	P	P	R1
26	GA-26	GA	P	P	P	R1

27	GA-27	GA	P	N	P	R2
28	GA-28	GA	P	N	P	R2
29	FL-1	FL	P	N	P	R2
30	FL-2	FL	P	P	P	R1
31	FL-3	FL	P	P	N	R3
32	FL-4	FL	P	N	P	R2
33	FL-5	FL	P	P	P	R1
34	FL-6	FL	P	P	P	R1
35	FL-7	FL	P	P	P	R1
36	FL-8	FL	P	P	P	R1
37	FL-9	FL	P	P	P	R1
38	FL-10	FL	P	P	P	R1
39	FL-11	FL	P	P	P	R1
40	FL-12	FL	P	P	N	R3
41	FL-13	FL	P	P	P	R1
42	FL-14	FL	P	P	P	R1
43	FL-15	FL	P	P	P	R1
44	FL-16	FL	P	P	P	R1
45	FL-17	FL	P	N	P	R2
46	FL-18	FL	P	P	P	R1
47	FL-19	FL	P	P	P	R1
48	FL-20	FL	P	P	P	R1
49	FL-21	FL	P	P	P	R1
50	FL-22	FL	P	N	P	R2
51	FL-23	FL	P	P	P	R1
52	FL-24	FL	P	P	P	R1
53	FL-25	FL	P	P	P	R1
54	FL-26	FL	P	P	P	R1
55	FL-27	FL	P	P	P	R1
56	FL-28	FL	P	P	N	R3
57	FL-29	FL	P	N	P	R2
58	FL-30	FL	P	P	P	R1
59	FL-31	FL	P	N	P	R2
60	FL-32	FL	P	P	P	R1
61	FL-33	FL	P	P	P	R1
62	FL-34	FL	P	P	P	R1
63	FL-35	FL	P	P	N	R3
64	FL-36	FL	P	P	P	R1
65	FL-37	FL	P	P	P	R1
66	FL-38	FL	P	P	P	R1
67	FL-39	FL	P	P	P	R1
68	FL-40	FL	P	P	N	R3
69	FL-41	FL	P	P	P	R1
70	FL-42	FL	P	P	N	R3
71	FL-43	FL	P	N	P	R2
72	FL-44	FL	P	P	P	R1

73	FL-45	FL	P	N	P	R2
74	FL-46	FL	P	N	P	R2
75	FL-47	FL	P	N	P	R2
76	FL-48	FL	P	P	P	R1
77	SC-1	SC	P	P	N	R3
78	SC-2	SC	P	P	P	R1
79	SC-3	SC	P	P	P	R1
80	SC-4	SC	P	P	P	R1
81	SC-5	SC	P	P	P	R1
82	SC-6	SC	P	P	P	R1
83	SC-7	SC	P	P	P	R1
84	SC-8	SC	P	P	P	R1
85	SC-9	SC	P	P	P	R1
86	SC-10	SC	P	N	P	R2
87	SC-11	SC	P	P	P	R1
88	SC-12	SC	P	N	P	R2
89	SC-13	SC	P	P	P	R1
90	SC-14	SC	P	N	P	R2
91	SC-15	SC	P	N	P	R2
92	SC-16	SC	P	N	P	R2
93	SC-17	SC	P	N	P	R2
94	SC-18	SC	P	N	P	R2
95	SC-19	SC	P	N	P	R2
96	SC-20	SC	P	P	P	R1
97	SC-21	SC	P	N	P	R2
98	SC-22	SC	P	N	P	R2
99	SC-23	SC	P	N	P	R2
100	SC-24	SC	P	P	N	R3
101	SC-25	SC	P	N	P	R2
102	SC-26	SC	P	N	P	R2
103	SC-27	SC	P	N	P	R2
104	SC-28	SC	P	N	P	R2
105	SC-29	SC	P	P	N	R3
106	SC-30	SC	P	P	N	R3
107	SC-31	SC	P	P	P	R1
108	SC-32	SC	P	P	P	R1
109	SC-33	SC	P	N	P	R2
110	SC-34	SC	P	P	P	R1
111	SC-35	SC	P	P	P	R1
112	SC-36	SC	P	N	P	R2
113	SC-37	SC	P	N	P	R2
114	SC-38	SC	P	N	P	R2
115	SC-39	SC	P	N	P	R2
116	SC-40	SC	P	P	P	R1
117	SC-41	SC	P	P	P	R1
118	SC-42	SC	P	P	P	R1

119	SC-43	SC	P	P	N	R3
120	SC-44	SC	P	P	N	R3
121	SC-45	SC	P	P	N	R3
122	SC-46	SC	P	P	P	R1
123	SC-47	SC	P	P	P	R1
124	SC-48	SC	P	P	N	R3
125	SC-49	SC	P	P	P	R1
126	SC-50	SC	P	P	N	R3
127	SC-51	SC	P	P	N	R3
128	SC-52	SC	P	P	N	R3
129	SC-53	SC	P	P	N	R3
130	SC-54	SC	P	P	P	R1
131	SC-55	SC	P	P	P	R1
132	SC-56	SC	P	P	N	R3
133	SC-57	SC	P	P	P	R1
134	SC-58	SC	P	P	N	R3
135	SC-59	SC	P	P	N	R3
136	SC-60	SC	P	P	P	R1
137	SC-61	SC	P	P	N	R3
138	SC-62	SC	P	P	N	R3
139	SC-63	SC	P	N	P	R2
140	SC-64	SC	P	P	P	R1
141	SC-65	SC	P	P	P	R1
142	SC-66	SC	P	P	P	R1
143	SC-67	SC	P	P	P	R1
144	SC-68	SC	P	P	P	R1
145	SC-69	SC	P	P	N	R3
146	SC-70	SC	P	P	P	R1
147	SC-71	SC	P	P	N	R3
148	SC-72	SC	P	P	P	R1
149	SC-73	SC	P	P	P	R1
150	SC-74	SC	P	P	N	R3
151	SC-75	SC	P	P	N	R3
152	SC-76	SC	P	P	N	R3
153	SC-77	SC	P	N	P	R2
154	SC-78	SC	P	P	P	R1
155	SC-79	SC	P	N	P	R2
156	SC-80	SC	P	N	P	R2
157	SC-81	SC	P	P	P	R1
158	SC-82	SC	P	P	N	R3
159	SC-83	SC	P	P	P	R1
160	SC-84	SC	P	P	P	R1
161	SC-85	SC	P	P	P	R1

Race determination was made according to the flowchart. P and N indicates positive and negative PCR amplification, respectively. R1 = Race 1, R2 = Race 2, R3 = Race 3.

Supplementary Table S2. List of samples bioassayed and submitted for molecular evaluation.

Sl #	Isolate Name	Bioassay results	Molecular results
1	FL-1	R2	R2
2	FL-2	R3	R1
3	FL-4	R2	R2
4	FL-5	R3	R1
5	FL-6	R1	R1
6	FL-7	R3	R3
7	FL-8	R1	R1
8	FL-9	R3	R1
9	FL-10	R2	R2
10	FL-11	R3	R1
11	FL-12	R3	R3
12	FL-13	R2	R1
13	FL-14	R3	R1
14	FL-15	R3	R3
15	FL-17	R2	R2
16	FL-18	R3	R1
17	FL-21	R3	R1
18	FL-22	R2	R2
19	FL-23	R1	R1
20	FL-25	R3	R3
21	FL-27	R1	R1
22	FL-28	R3	R3
23	FL-29	R2	R2
24	FL-30	R2	R1
25	FL-31	R2	R2
26	FL-33	R1	R1
27	FL-34	R3	R1
28	FL-35	R2	R3
29	FL-36	R2	R1
30	FL-37	R1	R1
31	FL-38	R2	R1
32	FL-40	R3	R3
33	FL-42	R3	R3
34	FL-43	R2	R2
35	FL-46	R2	R2
36	SC-1	R1	R3
37	SC-2	R1	R1
38	SC-3	R1	R1
39	SC-4	R1	R1
40	SC-5	R1	R1
41	SC-6	R1	R1
42	SC-7	R1	R1
43	SC-8	R1	R1

44	SC-9	R1	R1
45	SC-10	R2	R2
46	SC-11	R1	R1
47	SC-12	R2	R2
48	SC-13	R1	R2
49	SC-14	R2	R2
50	SC-15	R1	R2
51	SC-16	R2	R2
52	SC-17	R2	R2
53	SC-18	R2	R2
54	SC-19	R2	R2
55	SC-20	R2	R1
56	SC-21	R2	R2
57	SC-22	R2	R2
58	SC-23	R2	R2
59	SC-25	R2	R2
60	SC-27	R2	R2
61	SC-31	R1	R1
62	SC-32	R1	R1
63	SC-33	R2	R2
64	SC-34	R1	R1
65	SC-35	R1	R1
66	SC-36	R2	R2
67	SC-37	R2	R2
68	SC-39	R2	R2
69	SC-40	R1	R1
70	SC-41	R1	R1
71	SC-42	R2	R1
72	SC-46	R1	R1
73	SC-57	R1	R1
74	SC-70	R1	R1
75	SC-77	R2	R2
76	SC-78	R2	R1
77	SC-79	R2	R2
78	SC-80	R2	R2
79	GA-1	R2	R2
80	GA-2	R2	R2
81	GA-3	R2	R2
82	GA-4	R3	R3
83	GA-5	R3	R2
84	GA-6	R3	R3
85	GA-7	R3	R3
86	GA-9	R3	R3
87	GA-13	R1	R1
88	GA-14	R1	R1
89	GA-20	R3	R3

90	GA-23	R3	R3
91	GA-24	R3	R3
92	GA-27	R2	R2
93	GA-28	R2	R2
